# Cyclooxygenase-2 in Endometriosis

**DOI:** 10.7150/ijbs.35128

**Published:** 2019-10-23

**Authors:** Zhen-Zhen Lai, Hui-Li Yang, Si-Yao Ha, Kai-Kai Chang, Jie Mei, We-Jie Zhou, Xue-Min Qiu, Xiao-Qiu Wang, Rui Zhu, Da-Jin Li, Ming-Qing Li

**Affiliations:** 1 NHC Key Lab of Reproduction Regulation (Shanghai Institute of Planned Parenthood Research), Hospital of Obstetrics and Gynecology, Fudan University, Shanghai 200080, People's Republic of China.; 2 Department of Gynecology, Hospital of Obstetrics and Gynecology, Fudan University, Shanghai 200011, People's Republic of China.; 3 Reproductive Medicine Center, Department of Obstetrics and Gynecology, Nanjing Drum Tower Hospital, The Affiliated Hospital of Nanjing University Medical School, Nanjing, Jiangsu 210008, People's Republic of China.; 4 Clinical and Translational Research Center, Shanghai First Maternity and Infant Hospital, Tongji University School of Medicine, Shanghai 201204, People's Republic of China.; 5 Center for Human Reproduction and Genetics, Suzhou Municipal Hospital, Suzhou 215008, People's Republic of China.; 6 Shanghai Key Laboratory of Female Reproductive Endocrine Related Diseases, Hospital of Obstetrics and Gynecology, Fudan University, Shanghai 200011, People's Republic of China.

**Keywords:** COX-2, PGE_2_, endometriosis, pain, estrogen

## Abstract

Endometriosis (EMS) is the most common gynecological disease in women of reproductive age, and it is associated with chronic pelvic pain, dyspareunia and infertility. As a consequence of genetic, immune and environmental factors, endometriotic lesions have high cyclooxygenase (COX)-2 and COX-2-derived prostaglandin E_2_ (PGE_2_) biosynthesis compared with the normal endometrium. The transcription of the PTGS2 gene for COX-2 is associated with multiple intracellular signals, which converge to cause the activation of mitogen-activated protein kinases (MAPKs). COX-2 expression can be regulated by several factors, such as estrogen, hypoxia, proinflammatory cytokines, environmental pollutants, metabolites and metabolic enzymes, and platelets. High concentrations of COX-2 lead to high cell proliferation, a low level of apoptosis, high invasion, angiogenesis, EMS-related pain and infertility. COX-2-derived PGE_2_ performs a crucial function in EMS development by binding to EP2 and EP4 receptors. These basic findings have contributed to COX-2-targeted treatment in EMS, including COX-2 inhibitors, hormone drugs and glycyrrhizin. In this review, we summarize the most recent basic research in detail and provide a short summary of COX-2-targeted treatment.

## Introduction

Endometriosis (EMS) is a chronic gynecological disease that can usually be seen in women of reproductive age, and is characterized by the presence, transfer and invasion of functional endometrial tissue outside of the uterine cavity 1. Some hypotheses, such as retrograde menstrual reflux 2, ectopic presence of endometrial stem cells (ESCs) 3 and defects in the immune system 4, have been proposed to explain the migration, implantation and survival of the ectopic endometrial tissue and stroma. The incidence rate of EMS is 5-15% of all women of reproductive age and 20-50% of all infertile women 5-7, and the quality of life for endometriosis patients is significantly reduced, due to the increase in symptoms including chronic pelvic pain, dyspareunia, and infertility in comparison with women without EMS 8. The economic impact of EMS is compounded by the latency in the diagnosis of EMS, especially in young women who delay seeking treatment. The diagnosis of EMS is typically delayed by 8-10 years, because of the common misdiagnoses of EMS-induced pelvic pain as menstrual-related abdominal pain 9. EMS can be confirmed by direct visualization using laparoscopy and biopsy. In the past few years, the field of diagnostic biomarkers for EMS has gained increasing attention 10. When considering the theory of retrograde menstrual reflux, a puzzle emerges in that only around one tenth of women develop EMS, whereas retrograde menstruation is observed in most women, suggesting that other factors may also trigger the formation of endometriotic lesions, such as hormones, inflammatory factors, growth factors, angiogenic factors and cancer-related molecules 11.

The cyclooxygenase-2 (COX-2) / prostaglandin E_2_ (PGE_2_) pathway is closely related to EMS. There has been a general realization that EMS is a chronic pelvic inflammatory state, characterized by rising numbers of activated peritoneal immune cells, such as macrophages, and pro-inflammatory factors 12-14. COX-2 is thought to play a significant role in the origin and development of EMS 15. In endometrial and endometriotic tissues of women with EMS, elevated expression of COX-2 has also been described 16. COX-2 which is a rate-limiting enzyme in the PGE_2_ compound 17, is overexpressed in endometriotic tissues and contributes to increased concentrations of PGE_2_ in EMS patients, which have also been found in the peritoneal fluid (PF), as well as leukotrienes. COX-2/PGE_2_ signaling biologically activate oxygenated fatty acids, eicosanoids, and has been shown to be involved in various inflammatory pathological process 18. In EMS, they appear to play an important role in disease-associated pain 19, 20, essentially being the target of non-steroidal anti-inflammatory drugs (NSAIDs) 16. These inflammatory mediators, particularly COX-2/PGE_2_, may also be directly implicated in the pathogenesis of EMS 16, including the regulation of ectopic implantation and the growth of the endometrium, angiogenesis and immunosuppression 21. PGE_2_ is a major regulator of the immune response and can exert two opposing functions, exerting inflammatory or anti-inflammatory effects 22. Therefore, this paper systematically reviews the elements affecting the level and role of, and targeted drugs for COX-2 in EMS.

### COX-2

The enzyme COX was first demonstrated to exist in 1976 and cloned in 1988 23. COX has three isoforms: COX-1, COX-2 and COX-3 24-26. Among these, the COX-1 and COX-2 isoforms are often studied, due to the fact that they are associated with physiological as well as pathological processes. In the gastrointestinal and cardiovascular system, COX-1, a constitutively expressed house-keeping isozyme, is responsible for the basal production of essential PGs 27 that mediate homoeostatic functions. COX-3 is encoded by the COX-1 gene with reserve intron 1 in its mRNA. COX-3 is only expressed in some specific parts of the cerebral cortex and heart, and its exact functions are still unclear 28. The COX-2 isozyme, by contrast, is synthesized at very low levels under normal conditions and can be induced to become over-expressed under pathological conditions. The *PTGS2*, the gene for COX-2, is located on human chromosome 8 29. The promotor of the immediate-early gene *PTGS2* contains a TATA box and binding sites for several transcription factors, including nuclear factor-κB (NF-κB), the cyclic AMP response element binding protein (CRE), and the nuclear factor for interleukin-6 expression (*NF-IL-6*) 30, 31. COX-2 expression is associated with multiple transcriptional pathways. There is accumulating evidence for the critical involvement of COX-2 in various pathological processes that include inflammation 32, 33, cancer 34-36, neurodegenerative diseases 37, 38 and multidrug resistance 39.

The expression of COX-2 is rapidly upregulated in response to diverse pro-inflammatory and pathogenic stimuli. All signals converge upon the activation of mitogen-activated protein kinases (MAPKs) that regulate COX-2 expression at both the transcriptional and post-transcriptional levels 40. Lipopolysaccharide (LPS) signaling, the most pro-inflammatory mediators, induces the expression of COX-2 in the periphery. Specifically, LPS and other Toll-like receptor (TLR) ligands bind to MyD88-associated receptors and activate MEK/ERK pathway to induce the transcription factor activator protein 1 (AP1). LPS also can induce gene *PTGS2* transcription by activating the TRAF6/NIK/Tpl2/IKK/NF-κB pathway 41, 42. Nitric oxide (NO) affects the transcription of *PTGS2* in direct and indirect ways; directly, by increasing its catalytic activity, and indirectly, by triggering several signaling cascades that affect the gene transcription. NO and reactive oxygen species (ROS) increase *PTGS2* expression 43 via β-catenin/TCF pathway-mediated activation of *polyoma enhancer activator 3 (PEA3)* 44. Furthermore, several cytokines, including NO, several pro-inflammatory cytokines (e.g. IL-1, IFN-γ) and hypoxia inducible factor-1α (HIF-1α) can induce COX-2 expression through the cAMP/PKA/CREB and JNK/Jun/ATF2 signaling cascades 45-48. Growth factors can induce COX-2 expression in both normal and cancer cells, including insulin-like growth factor (IGF), transforming growth factor-α (TGF-α) and epidermal growth factor (EGF). Notably, this regulatory effect of IGF is mediated by PI3K and Src/extracellular signal-regulated kinase (ERK), while the effects of TGF and EGF are achieved through p38MAPK, ERK1/2 and PI3K 49. There are negative regulators for COX-2 expression. For example, glycogen synthase kinase 3 (GSK3) suppresses COX-2 expression through inhibition of the β-catenin/transcription factor-4 (TCF4) and PKCδ/ERK1/2 signaling pathways 50.

### COX-2 expression in EMS

COX-2 is mainly expressed in the endometrial glandular epithelium in healthy women and varies during the menstrual cycle. The expression of COX-2 is at its lowest in the early proliferative phase and gradually increased thereafter, and it maintains a high level throughout the secretory phase 51. In women with EMS, the expression of COX-2 in the endometrial glandular epithelium, endometrial stroma 4 and PF was higher than that in the control group 52, and it also varies throughout the menstrual cycle 53. Cho *et al.* 54 demonstrated that in EMS patients, the expression of COX-2 was elevated significantly in the eutopic endometrium during the proliferative phase and in ovarian endometriotic tissue during the secretory phase compared with the control groups. In addition, ectopic lesions highly express COX-2 in endometriosis patients with chronic stress 54. Notably, mRNA expression of *PTGS2* in the endometrium and ovarian lesions significantly correlates with serum CA-125 and the diameter of endometriomas 54. In recent research, Mei *et al.* [55]found that the number of COX-2^+^CD16^-^ NK cells with impaired cytotoxic activity in the abdominal cavity fluid of patients with EMS was markedly higher than that of the control group.

### Genetic variation in *PTGS2* (COX-2) and the risk of EMS

Gene polymorphisms in *PTGS2* are associated with a high risk of many diseases, such as EMS 56, cancer 57, and acute pancreatitis 58. The cloning, sequencing and expression of human *PTGS2* cDNA have been previously described 59. There are 51 CpG sites in the promoter region of the COX-2 gene from -590 to +186. Three main transcription factors predominantly regulate COX-2 expression, including *NF-κB*, *NF-IL6*, and* CRE* 60, 61. Moreover, in many cancers, aberrant methylation of promoter CpG island of the COX-2 has been regarded as an alternative mechanism of its abnormal expression and contributes to carcinogenesis 62, 63. Genes associated with endometriosis have abnormal DNA methylation. Wang *et al* [64]and Zidan *et al* 56 reported that DNA hypomethylation of the *NF-IL6* site within the promoter of the *PTGS2* gene was highly correlated with the pathological process of EMS, suggesting that EMS may be an epigenetic disease. Wang *et al.* 64 found that *PTGS2* genotypic frequencies of G to A at the -1195 locus in the promoter region in EMS were significantly different from those in normal women. Moreover, the allele frequency in EMS was markedly higher than that in normal women. The risk of EMS for those carrying two A alleles was 2.19 and 2.41 times greater than that for the to non-A genotype. In addition, Wang *et al.* 65 demonstrated that on the promoter region of the *PTGS2* gene, the -1195 A/G may increase the risk of pain occurrence in women with EMS. The presence of the ancestral allele, -765G/C, of the *PTGS2* gene is associated with an increased risk of pathological progression in moderate/severe EMS which is related to fertility, and the expression of *COX*-*2 in the* eutopic endometrium of women with EMS has shown a tendency to increase when compared to the control group 66, 67. In a Korean study, the -765C allele was a protective agent against the development of the disease 68.

### Regulation of COX-2 expression

**Figure 1:**
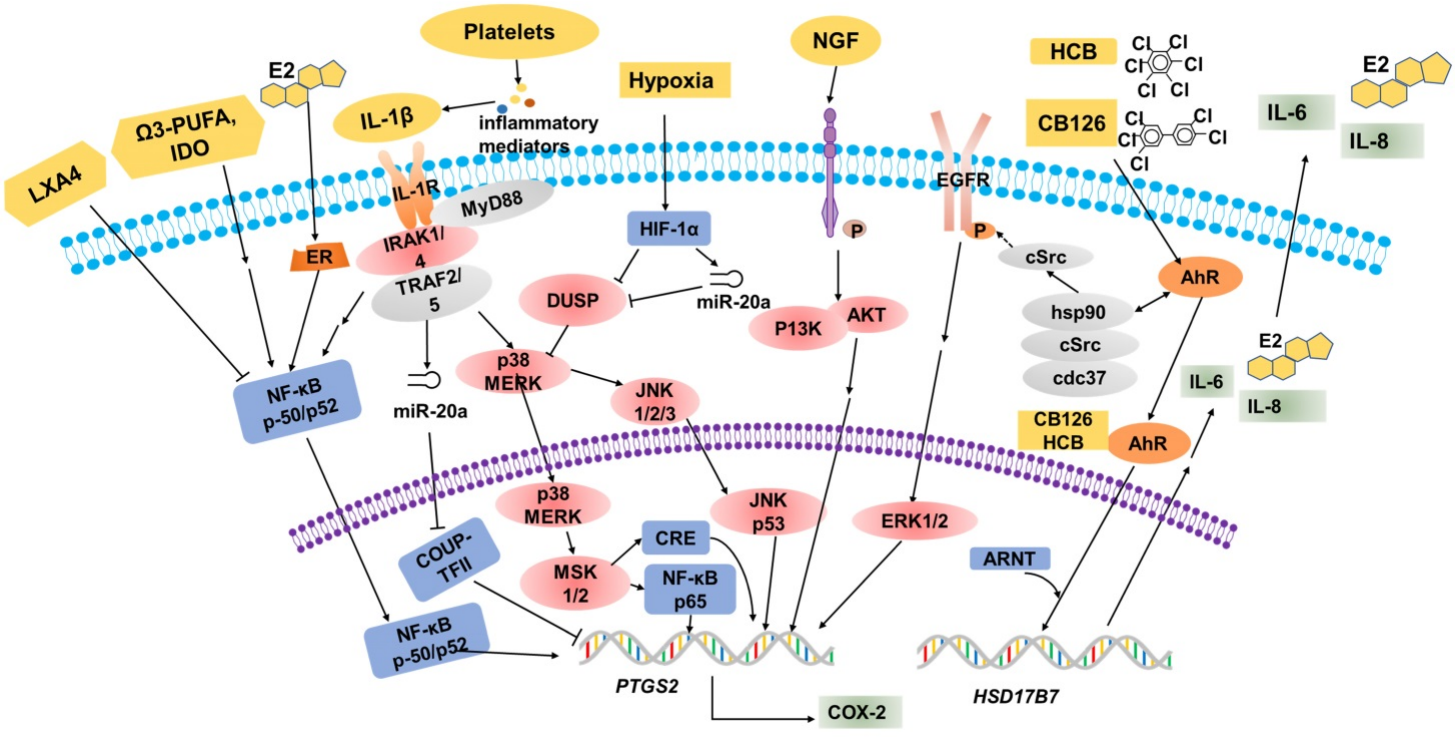
** Multiple factors regulate COX-2 expression.** Estrogen (E_2_), omega-3 PUFA and IL-1β promote COX-2 expression through the NF-κB signaling pathway. IL-1β stimulates the phosphorylation of MERK, p38 and JNK, then CRE and NF-κB p65 bind to sites on the COX-2 promoter to increase COX-2 expression. In hypoxic conditions, activated HIF-1α will suppress DUSP2 expression directly, and then result in the hypersensitivity of COX-2 in response to proinflammatory stimuli (e.g. IL-1β). Elevated NGF markedly upregulates the expression of PTGS2/COX-2 via the PI3K/AKT signaling pathway. Environmental pollutants, for example HCB and CB126, are known to act via the AhR. These organic compounds have some biological effects mediated by the activation of the cytosolic AhR complex (AhR-dioxin-c-Src), and regulate PTGS2 transcription indirectly. The combination of organic compounds and AhR induces HSD17B7 expression and results in the upregulation of E_2_, IL-6 and IL-8, which will further promote COX-2 expression.

Over the years, many epidemiological, pharmacological and laboratory studies have demonstrated that various factors are involved in the regulation of COX-2 expression in EMS (**Table 1, Figure 1**).

### Estrogen

Estradiol and progesterone are core hormones regulating the function of endometrial tissue. In the course of different phases of the menstrual cycle, each steroid hormone is estimated to regulate the translation of hundreds of genes successively 15, 69. Ectopic and eutopic endometrial tissues have apparently similar histological changes in response to estradiol and progesterone, and both tissues express immunoreactive estrogen and progesterone receptors (PRs). This locally produced estrogen in both the ectopic and eutopic endometrium is considered to exert a crucial role in the regulation of the immunological mechanisms responsible for controlling the development of EMS 15. Local estrogen production hastens prostaglandin synthesis by stimulating COX-2 activity, thus creating a positive feedback loop of augmented estrogen formation and enhanced inflammation. The synthesis of proinflammatory PGs such as COX-2-derived PGE_2_, can be activated by NF-κB and increased by estrogen in the endometrium 70. The synthesis of aromatase seems to play a pivotal role in the development of EMS, which is stimulated by PGs and other inflammatory mediators in endometrial cells but not in aromatase-negative endometrial cells 71. Thus, a large amount of local estrogen production will further enhance PG synthesis by activating COX-2 expression.

### Proinflammatory Cytokines

It has been reported that ectopic ESCs are hypersensitive to the stimulating effect of cytokines, such as interleukin-1β (IL-1β), in terms of overexpression of COX-2 46. IL-1β can accelerate the synthesis of COX-2 at the mRNA, protein, and enzyme activity levels in a model system of EMS. Notably, IL-1β can activate MAPK-dependent signaling by binding to the CRE site at -571/-564 of the COX-2 promoter to increase IL-1β-induced COX-2 expression 46. COX-2 gene induction by IL-1β involves the ERK1/2 and NF-κB signaling pathway, because IL-1β stimulates the phosphorylation of ERK, p38 and JNK 72-74. Nerve growth factor (NGF), a core endocrine regulator for the growth of neurons, plays crucial roles in the regulation of neuronal survival and maturation 75. In inflamed tissues in numerous diseases, overexpressed NGF regulates immune responses; directly or indirectly: directly, by influencing innate and adaptive immune responses, and indirectly inducing the release of immune-active neuropeptides and neurotransmitters^76^. NGF is believed to contribute to pathological pain associated with various medical conditions, such as cancer and rheumatoid arthritis (RA) 77. Elevated NGF levels markedly increase the expression of *PTGS-2*/COX-2 at the mRNA and protein levels as well as PGE_2_ secretion in women with EMS. This association may be regulated by enhanced nerve bundle density and by COX-2/PGE_2_ stimulation via the high-affinity Trk receptor 78-80.

### Hypoxia

Hypoxia, which plays a key role in immunity and inflammation under both physiological and pathological conditions, arises when cellular oxygen demand exceeds supply 81. Hypoxia triggers a profound change in gene transcription, and hypoxia-inducible factor (HIF) is a master regulator 82. HIF-1α is one of the major transcriptionally active isoforms of HIF that have been described 83. Dual-specificity phosphatase-2 (DUSP2) which is a nuclear phosphatase that can specifically dephosphorylate p38 MAPK and ERK 84, is markedly downregulated in stromal cells of ectopic endometriotic tissues, leading to prolonged activation of p38 MAPK and ERK and increased COX-2 expression 85. HIF-1α suppresses DUSP2 expression directly, leads to sustained activation of p38 MAPK and ERK, and ultimately contributes to aberrant COX-2 synthesis in ectopic endometriotic stromal cells 86. The ERK and p38 MAPK signaling pathways have been reported to play important roles in the modulation of PGE_2_ synthesis in ectopic endometrial cells, and abnormal phosphorylation of ERK and/or p38 MAPK may lead to over-expression of COX-2 in ectopic lesions 45, 87. Down-regulation of hypoxia-mediated DUSP2 leads to more activated ERKs and p38 MAPK, and ultimately results in the hypersensitivity of COX-2 in response to proinflammatory stimuli. In addition, microRNAs (miRNAs) are related to tissue repair, hypoxia, inflammation, cell proliferation, extracellular matrix remodeling, apoptosis and angiogenesis in EMS 88. It has been demonstrated that the expression of miR-20a induced by hypoxia is relatively high in ectopic endometrial tissues compared to that in eutopic endometrial tissues 86, 89. Interestingly, DUSP2 is a target of miR-20a. A previous study suggested that hypoxia-induced miR-20a expression leads to downregulation of DUSP2 expression, and results in the overexpression of downstream ERK-regulated genes, such as angiogenic, and mitogenic factors, and COX-2 87. Taken together, these data strongly support the hypothesis that hypoxia is a vital factor that potentiates *PTGS2* gene sensitivity in ESCs 90.

### Environmental pollutants

During the last few years, increasing evidence has emerged in support of the relationship between exposure to chemicals with endocrine disruption potential and hormone-related gynecological diseases shows steadily increased 91. Environmental organochlorine pollutants, particularly polychlorinated biphenyls (PCBs) and dioxins, are thought to be involved in the development of EMS 94. Dioxin-like 92, 93 rather than non-dioxin-like PCB congeners 94 tend to be responsible for the pathological risk of EMS, according to current epidemiological evidences. Huang* et al.* 95 found that CB126 (a dioxin-like PCB) enhances estradiol (E2) biosynthesis and promotes the secretion of both IL-6 and IL-8. CB126 is known to act *via* the aromatic hydrocarbon receptor (AhR). Using DMF to inhibit this receptor can abolish the effects induced by CB126 96. The gene expression of HSD17B7, rather than aromatase (CYP19A) or HSD17B1, is up-regulated after exposure to CB126. For local E_2_ production in endometriotic lesions, CYP19A was previously thought to be significant 97, 98. The expression of HSD17B7 can be enhanced by LPS and IL-1β which can be observed in ESCs. Thus, the development of EMS can be promoted by the interaction between the endocrine and immune systems and CB126 may provoke this process through stimulation of both E2 synthesis and the inflammatory response. This may support the idea that PCB-induced EMS is related to COX-2. Another type of organochlorinated pollutant, hexachlorobenzene (HCB), is a “dioxin-like” organic compound that binds to AhR 99, accumulating in lipid tissue and inducing the synthesis of xenobiotic metabolic enzymes. These organic compounds have some biological effects which are mediated by the activation of the cytosolic AhR complex (AhR-dioxin-c-Src), triggering membrane actions where c-Src activates growth factor receptors, and nuclear actions where AhR regulates gene transcription including for COX-2 100, 101. Chiappini *et al* 102 found that exposure to HCB enhanced COX-2, PGE_2_ and EP4 expression, and c-Src kinase activation in T-HESC, thereby contributing to EMS development through both hormonal regulation and immune function.

### Metabolites and metabolic enzymes

*In vivo* and *in vitro* studies have demonstrated that omega-3 polyunsaturated fatty acids (omega-3 PUFAs) have potential antiapoptotic, anti-inflammatory, antiangiogenic, and antiproliferative effects 103. Omega-3 PUFAs block the activation of NF-κB, cut down the production of pro-inflammatory cytokines such as IL-6, TNF-α and IL-1, and reduce COX-2 expression to protect against the development of EMS 104, 105. In particular, the 12/15-LOX-pathway products of eicosatetraenoic acid (EPA) may be critical mediators in suppressing EMS^104^. In inflammatory bowel disease (IBD), PUFAs of the n-3 series have reported to exert an inhibitory action on *PTGS2* gene expression* in vivo* using a genetically-modified mouse 106; they compete with arachidonic acid (AA) for binding to the COX-2 catalytic site and finally obstructed prostaglandin formation 107. Indoleamine 2,3-dioxygenase (IDO) has the capacity of tryptophan consumption and the generation of proapoptotic metabolites, thus it was confirmed to be an immune modulator 108 and to be highly expressed in EMS-derived eutopic and ectopic ESCs; it also upregulates COX-2 expression by means of the activation of the JNK signaling pathway 109, 110, along with the enhancement of cell survival, proliferation and invasion. In the canonical JNK pathway, activated JNK can lead to phosphorylation of the transcriptional activation domain of c-Jun; this phosphorylated domain constitutes AP-1, a kind of transcription factors which is acted on the human IDO gene promoter region 111, with c-Fos 112. Subsequently, G protein-coupled receptors regulate MAPK signaling pathways that result in specific response gene expression, including the genes associated with cell proliferation, apoptosis and invasion 113. Lipoxins are endogenous eicosanoids, generally produced via a transcellular biosynthetic pathway, the functions of which exhibit both pro-resolving and anti-inflammatory properties 114. *In vivo studies,* Lipoxin A_4_ (LXA_4_) mediates anti-inflammatory activities through multiple receptors*115*, and the best characterized lipoxin A4 receptors is (ALX)/formyl peptide receptor 2 (FPR2). LXA_4_ treatment significantly attenuated COX-2 and PGE_2_ levels in both endometriotic lesions and peritoneal fluid cells, which might be the result of downregulating CYP19a1 expression or via direct transcriptional repression 116.

### Platelets

Inflammation and coagulation are intricately entwined: inflammation stimulates the coagulation cascade and coagulation modulates the inflammatory response in many ways 117, 118. Platelets are aggregated in endometriotic lesions, concomitantly with the elevated levels of VEGF and microvessel density. A co-culture system of endometriotic stromal cells and platelets led to enhanced cellular proliferation, and increased COX-2 expression. Analysis of the underlying mechanisms demonstrated that platelet granules contain a variety of inflammatory mediators, such as, IL-1β, which induce the expression of COX-2 in a dose-dependent manner in both normal ESCs and ectopic ESCs 119.

### Others

Chicken ovalbumin upstream promoter-transcription factor II (COUP-TFII, also known as NR2F2) is an orphan nuclear receptor that has a pivotal impact in embryonic implantation and placentation, indicating that it is a key regulator in uterine physiology 120-122. In normal and endometriotic stroma, the expression levels of COUP-TFII mRNA and protein have been dentified to be different, which highlights its potential functions in endometriotic pathogenesis. In normal endometrial tissue, COUP-TFII directly binds to the *PTGS2* promoter to inhibit its transcription and diminish IL-1β-induced COX-2 over-expression 123. In endometriotic stroma, cytokines IL-1β, TNF-α and TGF-β1 can repress COUP-TF II expression mediated by miR-302a, then suppress the binding of COUP-TFII to the COX-2 promoter 123. Therefore, the decreased COUP-TFII results in the derepression of COX-2 in ESCs 124. However, the detailed mechanism requires further research.

## The role of COX-2 in EMS

### Cell proliferation and apoptosis

**Figure 2:**
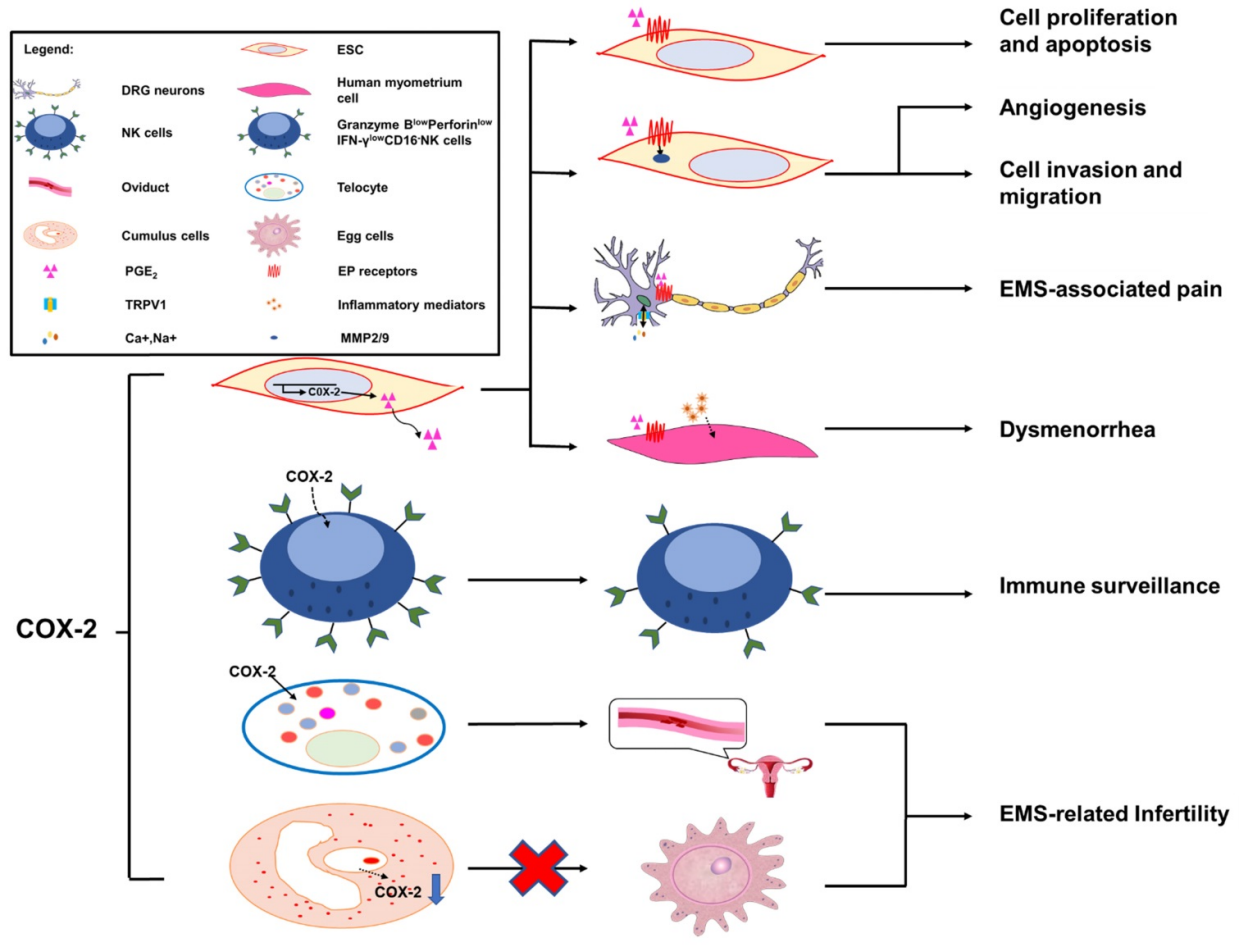
** The role of COX-2 in EMS.** Overexpression of COX-2 has been demonstrated to be a master regulator in the progression of endometriosis. A high level of COX-2 can promote cell proliferation and suppress cell apoptosis via trans-activating multiple complex signaling pathways, which are triggered by PGE_2_ and its receptors, EP2 and EP4. In addition, MMP-2/9 activity regulated by PGE_2_ is be involved in angiogenesis, and ESC migration and invasion, via the intracellular MAPK, AKT and Wnt signaling pathways. COX-2 can induce COX-2^+^CD16^-^NK cell (Granzyme B^low^Perforin^low^IFN-γ^low^CD16-NK cell) differentiation in the peritoneal fluids of patients with endometriosis, which is beneficial to the immune escape of endometriotic lesions. The COX-2/PGE_2_/EP2-EP4 signaling decreases the threshold and enhances the excitability of nociceptor sensory fibers through TRPV1 and SCN11A, and contributes to EMS-associated pain. A high level of COX-2/PGE_2_ and COX-2-induced inflammatory mediators increase uterine tone and contractions and cause pain. TCs are important in maintaining the structural and reproductive functional normality of the oviduct, while overproduced COX-2 may damage the functions of TCs, which will lead to infertility. The low production of COX-2 in cumulus cells is regarded as a possible mechanism of EMS-related infertility.

The growth of endometriotic lesions is a process tightly regulated by a delicate balance between proliferation and apoptosis in endometrial cells. This abnormal survival ability has been associated with the concomitant overproduction of antiapoptotic factors and underproduction of proapoptotic factors 125. As shown in **Figure 2**, COX-2-induced PGE_2_ is a significant antiapoptotic mediator; it can activate cell survival and antiapoptotic pathways to prevent cells from undergoing programmed cell death or apoptosis. The binding of PGE_2_ and its receptors, EP2 and EP4, regulates these complex molecular interactions and promotes the survival of human ESCs outside the uterus via multiple trans-activating complex signaling pathways (such as c-Src/β-arrestin 1/EGFR/ERK1/2, c-Src/βarrestin1/TNFαR1, IL-1βR1/IκB/NFκB or Gsα/axin/β-catenin)^128^. Selective inhibitors of EP2 and EP4 impair ESC survival pathways and facilitate interactions between antiapoptotic proteins (Bcl-2/Bcl-XL) and proapoptotic proteins (Bax/Bad) leading to an augmentation of the release of cytochrome c and activation of the caspase-3/PARP pathways 126. The results indicated that administration of NS-398, a kind of selective COX-2-inhibitor, and siRNA can significantly reduce COX-2 concentration, PGE_2_ production, and endometriotic epithelial and stromal cell proliferation 127. Laschke *et al*. 127 showed that in an EMS mouse model, treatment with NS-398 applied to endometrial grafts led to a tendency towards decreased cell proliferation, along with a sustained reduction in proliferating cell nuclear antigen (PCNA) expression; in addition, an increased number of apoptotic cells was observed, as indicated by an upregulation of activated caspase-3. Furthermore, epithelial cell lines stably transfected to overexpress the PTGS2 gene appear to have a higher proliferation rate and to inhibit apoptosis by means of reacting with cyclin D to elongate the G1 phase of the cell cycle 128, 129. Therefore, the administration of selective COX-2 inhibitors to the ectopic and eutopic endometrium may contribute to an inhibition in proliferative potential and a growth rate in apoptosis 130.

### Cell invasion and migration

PGE_2_ exerts its biological effects through G protein-coupled receptors and by activating multiple cell signaling pathways. These G protein-coupled receptors are designated according to the four subtypes of the PGE receptor (EP1, EP2, EP3 and EP4) 131. Previous studies have illustrated that EP receptors intracellularly trans-activate the MAPK, AKT and Wnt signaling pathways, resulting in the modulation of cell apoptosis, proliferation, invasion, migration, angiogenesis, pain and immunomodulation 132, 133. Administration of COX-2 inhibitors decreases the survival, migration and invasion of endometriotic cells as a result of decreased production of PGE_2_ 127, 134. Additionally, COX-2-associated migration and invasion are decreased when COX-2 is inhibited in endometriotic cells, and are mediated by matrix metalloproteinase (MMP)-2 and MMP-9 in humans 135. In addition, there is an interesting observation that COX-2 inhibitors produce more detrimental effects on invasion compared with migration in endometriotic cells; however, the underlying molecular mechanisms of these selective effects are unknown 21.

### Angiogenesis

In the pathological process of EMS, the development of new blood vessels represents a core factor, because the long-term survival and growth of the exfoliated endometrium requires an effective blood supply; this is a major prerequisite at ectopic lesions. The development of the ectopic endometrium relies on angiogenesis, which is a characterizing factor of EMS 48. MMPs, a group of zinc-dependent proteolytic enzymes, are mainly involved in extracellular matrix degradation to promote cellular invasion, migration and angiogenesis 136, 137. *In vitro*, some evidence suggests that PGE_2_ dramatically increases MMP-2 activity as well as tube formation 138. Blocking the expression of COX-2 and/or a phosphorylated protein kinase (AKT) suppresses MMP-2 activity and endothelial tube formation, indicating that the MMP-2 activity modulated by PGE_2_ is potentially involved in angiogenesis. Moreover, treatment with a chemical inhibitor can specifically inhibit MMP-2 by significantly inhibiting cellular migration, invasion and tube formation. Furthermore, a notable decrease in endometrial lesion numbers was observed after applying inhibitors of MMP-2 and COX-2 to the mouse model of EMS. Collectively, COX-2 can promote angiogenesis indirectly via the involvement of MMP-2 activity during EMS progression 138. In particular, COX-2 inhibitors could exert an anti-angiogenic effect on endometriotic lesions. On one hand, the angiogenic factor vascular endothelial growth factor (VEGF) plays an important role in the pathogenesis of EMS 48, and selective COX-2 inhibitors suppress the expression of VEGF in endometrial grafts initially 127 and in tumor researches 139. On the other hand, in a study on hamsters, firm platelet adhesion to the endothelium of microvessels was increased when treated with a selective inhibitor of COX-2 140.

### EMS-associated pain: chronic pelvic pain and dysmenorrhea

COX-2 is inducible and is involved in pain- and inflammation-associated pathological pathways 141. Increased expression levels of COX-2 in central nervous system (CNS) regions within the pain-processing pathway were found at the spinal 142, thalamic and cortical levels 143, and in dorsal root ganglion (DRG) neurons 144. COX-2 expression is viewed as a sensitive and responsive biomarker of centralized inflammatory pain in the CNS 142. In a rat EMS model, sympathetic and sensory C and Aδ fibers innervated endometriosis lesions, which expressed calcitonin gene related peptide (CGRP) and TRPV1 proteins, thereby contributing to the formation of the proinflammatory microenvironment of DRG neurons from L1-S1. Neurons from L1-S1 innervate the pelvis and pelvic organs and increase pelvic floor hyperalgesia 145. Greaves *et al* 143 found that in an EMS mouse model, the COX-2/PGE_2_ signaling pathway was overexpressed. PGE_2_ plays a significant role in the pathophysiology of COX-2-induced EMS 143. PGE_2_ acts on peripheral nociceptors, lowering the threshold and enhancing the excitability of nociceptor sensory fibers through TRPV1 and Nav1.9 voltage-gated sodium channels (SCN11A) 146, and induces chronic inflammatory pain through EP2 and EP4 147, 148. Localized peripheral inflammation increases the expression of EP4 protein in L5 DRG neurons. Inhibition of EP4 decreases the PGE_2_-induced sensitization of DRG neurons and the release of the neuropeptides SP and CGRP 147, 148. At the level of the PTSG2 gene, the -1195 A/G on the promoter region of the COX-2 gene may increase the risk of pain occurrence in patients with EMS, possibly by affecting the rate of gene expression, especially in patients with the pain phenotype 66.

Dysmenorrhea, defined as painful cramps in the lower abdomen that occurs with menstruation, is one of three main characteristics of EMS 149. Primary dysmenorrhea is one of two types of dysmenorrhea, caused by an increased or unbalanced level of endometrial prostaglandins, most importantly PGE_2_, during menstruation 150. COX-2-derived PGE_2_ increases uterine tone and contractions, and causes pain 151. COX-2 can induce the production of a large number of inflammatory mediators, including PGs 152, and contribute to dysmenorrhea in patients with EMS.

### EMS-related infertility

Around 20-50% of the EMS population is estimated to be infertile 153. Telocytes (TCs; previously considered to be interstitial Cajal-like cells, ICLC), a peculiar type of stromal cell, have been identified in many organs, including the endometrium, myometrium and fallopian tube 154, and have been reported to be decreased in women with EMS and tubal ectopic pregnancy 155. Structural and reproductive functional abnormalities of the oviduct are observed as a result of TC damage 156. In oviduct tissues, overproduced COX-2 may be responsible for the TC damage 157. The pathologic niche of EMS is considered to have deleterious effect on oocyte quality. Cumulus cells are indirect biomarkers of this 158-160. In eutopic and ectopic endometrial tissues from women with EMS, the transcription of PTGS2 is upregulated 15, 161, 162. By contrast, the transcript levels of PTGS2 in cumulus cells of infertile women with EMS are decreased 163. Reduced PTGS2/COX-2 expression may lead to an impairment of oocyte quality, which is regarded as a possible mechanism of EMS-related infertility 163.

### Immune surveillance

The transcription of the aromatase gene is favored by epigenetic changes in the endometrium, allowing endometrial cells to survive in ectopic locations by producing enough estrogen to protect them from destruction by activated macrophages 70. Local estrogen production accelerates PG synthesis by stimulating the activation of COX-2, thus creating a positive-feedback sequence of facilitated estrogen formation and enhanced inflammation 70. Therefore, the increased inflammation in EMS may reflect the overexpression of estrogen, which alone activates COX-2 and NF-κB to increase inflammation and PG production. In a recent study, a high level of COX-2^+^CD16^-^NK cells was observed in the peritoneal fluid of patients with EMS 55. COX-2 can induce the differentiation of low-cytotoxicity CD16^-^NK cells (with low levels of Granzyme B, Perforin and IFN-γ), and promote the immune escape of endometriotic lesions. In addition, these COX-2^+^CD16^-^NK cells promote the proliferation and restrict the apoptosis of ectopic lesions; however, the mechanism needs further study 23. The population of Foxp3+ regulatory T (Treg) cells is upregulated in the PF of EMS patients, which contributes to the local dysfunctional immune microenvironment in EMS and the immune escape of ectopic endometrial tissue. The estrogen-IDO1-MRC2 axis is involved in regulating the differentiation and function of Treg cells 164. It was reported that Treg cells upregulate the expression of MMP2 and COX-2 and promote the survival, migration and invasion of endometriotic cells 165. In the gastric tumor microenvironment, COX-2 expression is also strongly correlated with Foxp3, a reliable marker of Treg cells 166. Yuan X.Y *et al* 150 found that Treg cells could express high levels of COX-2, and produced a high level of PGE_2_. PGE2 binds to EP2 and EP4 and triggers the cAMP Csk inhibitory pathway to suppress T-cell immune responses. Foxp3^high^ Treg cells suppress the proliferation of autologous CD4^+^CD25^-^ T cells, which can be reversed by COX inhibitors and PGE_2_ receptor-specific antagonists. These data show that in the development of gastric cancer, tumor-infiltrating Treg cells can induce immune suppression via the COX-2/PGE_2_ axis 150,167.

### Anti-EMS drugs targeting COX-2 (Figure 3)

#### COX-2 inhibitors

COX-2 is an essential therapeutic target for anti-inflammatory drugs, which are known as nonsteroidal anti-inflammatory drugs (NSAIDs), including naproxen and diclofenac, as well as newer COX-2 selective inhibitors such as Celebrex (celecoxib; Pfizer). A clinical trial recruited 28 women (age range 23-39 years) who were diagnosed with EMS by laparoscopy. They were treated with a placebo or a COX-2 specific inhibitor. It was found that the administration of NSAIDs was safe and effective in the management of EMS-related pain and might block angiogenesis in endometriotic foci, which was considered to be a long-term effect in that it may help prevent relapses of EMS 168. In a rat model, a new selective COX-2 enzyme inhibitor, dexketoprofen trometamol, remarkably reduced the development of experimentally-induced endometriotic lesions, both macroscopically and microscopically 169, 170. COX-2 induces the production of PGE_2_ and E_2_, which are known to increase VEGF expression 171. The binding of VEGF to the Fms-like tyrosine kinase 1 (Flt-1) receptor 170 leads to an upregulation in mitogenesis, migration enhancement, and the release of various proteolytic enzymes. It has been demonstrated that treatment with parecoxib downregulates the expression of VEGF and Flk-1, and reinforces its antiangiogenic activity in rat endometriotic lesions 172. It was reported that patients with EMS showed increased numbers of activated macrophages in the PF 14, 173, which are the primary source of VEGF produced in areas of inflammation 14. Treatment with COX-2 inhibitors significantly decreases microvessel density and macrophage numbers, and is associated with decreased expression of VEGF and Flk-1 172, 174. In mouse model, the group administered with COX-2 inhibitors showed a low concentration of PGE_2_. Combined use of COX-2 inhibitors and telmisartan may be more effective in the treatment of endometriotic lesions. Combining the inhibition of COX-2 with the peroxisome proliferator-activated receptor (PPAR)-γ agonist telmisartan appears to be a promising strategy in EMS as it suppresses cell proliferation and induces apoptosis. Decreased expression of p-Akt/Akt and downstream p-eNOS/eNOS in parecoxib/telmisartan-treated lesions has also been shown experimentally 175. However, COX-2 inhibitors may damage the gastrointestinal tract, and induce the development of erosions and ulcers, with potential complications of protein loss, stricture formation, bleeding and perforation 176. The side effects of COX-2 inhibitors should be monitored.

#### Hormone drugs

Type-II gonadotropin releasing hormone (GnRH II), a secondary form of GnRH, is distributed in discrete regions of the central and peripheral nervous systems and in nonneural tissues; GnRH-II functions in the nervous system and, notably, in areas associated with sexual behavior 177. GnRH-II has the effect of promoting apoptosis, especially on the ectopic ESC, as a result of inhibiting the secretion of IL-8 protein and the level of COX-2 mRNA and IL-8 mRNA in endometriotic cells, and in the case of the downregulation of endogenous GnRH-II expression it can lead to the initiation and development of EMS 178. In addition, GnRH-II decreases VEGF secretion in the ectopic, eutopic and normal ESC in EMS *in vitro*, which contributes to the downregulation of the number of newly-formed blood vessels 177. The IL-1β-induced expression of COX-2 in ESC can be reversed by GnRH-II 179. Dienogest (DNG) is a selective progesterone receptor (PR) agonist. One of the current clinical anti-EMS strategies is oral administration of DNG. However, PR has been reported to appear as two major isoforms, PR-A and PR-B, and they have mostly distinct physiological functions 180. DNG exerts therapeutic efficacy against the pain and progression of EMS regardless of PR expression patterns. It was reported that DNG downregulates the mRNA expression of CYP19A1, COX-2, mPGES-1, IL-8, IL-6, monocyte chemoattractant protein (MCP)-1, VEGF and NGF, and PGE_2_ production in human endometriotic epithelial cell lines that specifically express either PR-A or PR-B 181, 182.

#### Other drugs

Glycyrrhizin, a triterpene isolated from the roots and rhizomes of licorice (*Glycyrrhiza glabra*), has been shown to have anti-inflammatory effects. Wang *et al* 182 found that glycyrrhizin was able to attenuate the expression of inducible nitric oxide synthase (iNOS) and COX-2 in mouse endometrial epithelial cells (MEECs). Furthermore, glycyrrhizin dramatically diminishes LPS-inducing TLR4 expression and NF-κB activation in MEECs. As a result, it can inhibit the LPS-induced inflammatory response. Glycyrrhizin may be used as a potential agent for the treatment of EMS, partly by targeting COX-2 183. Another traditional Chinese medicine, puerarin, extracted from* Radix puerariae*, is widely known as a natural conditioner of selective estrogen receptors (ERs) 184. Puerarin can inhibit the expression of P450arom and COX-2 in the ectopic endometrium, restrict the levels of E2 and PGE_2_, and block the positive feedback mechanism of E2 synthesis. It could be a potential therapeutic agent for the treatment of EMS in clinic 185.

### Conclusion and future perspectives

Under the regulation of hormone, hypoxia and so on, the increased COX-2 in the glandular epithelial cells and ESCs of ectopic lesions leads to the high proliferation, low level of apoptosis, high invasion and angiogenesis, and impaired cytotoxic NK cell differentiation, which further promotes the occurrence and development of EMS. By producing PGE_2_ to induce EMS-related pain, COX-2 in endometriotic cells can further accelerate the development of EMS. Many drugs and COX-2 inhibitors play an important role in the treatment of EMS by targeting COX-2, especially for EMS-related pain. However, further investigation of their actions, apart from analgesic functions, is needed, which will enlarge therapeutic horizon of these drugs in EMS. For example, considering the important role of COX-2 in the survival, invasion, angiogenesis and immune escape of ectopic lesions, COX-2 may be an important indicator for predicting the recurrence of EMS. Prophylactic drugs may become available in high-risk populations. COX-2-targeting treatments may inhibit the growth of the ectopic intima, relieve pain, reduce angiogenesis and remove residual lesions. By analyzing the expression level of COX-2 and the PGE_2_ concentration in the endometriotic microenvironment, there is potential to provide individualized and precise treatment for preventing the recurrence of EMS.

## Figures and Tables

**Figure 3:**
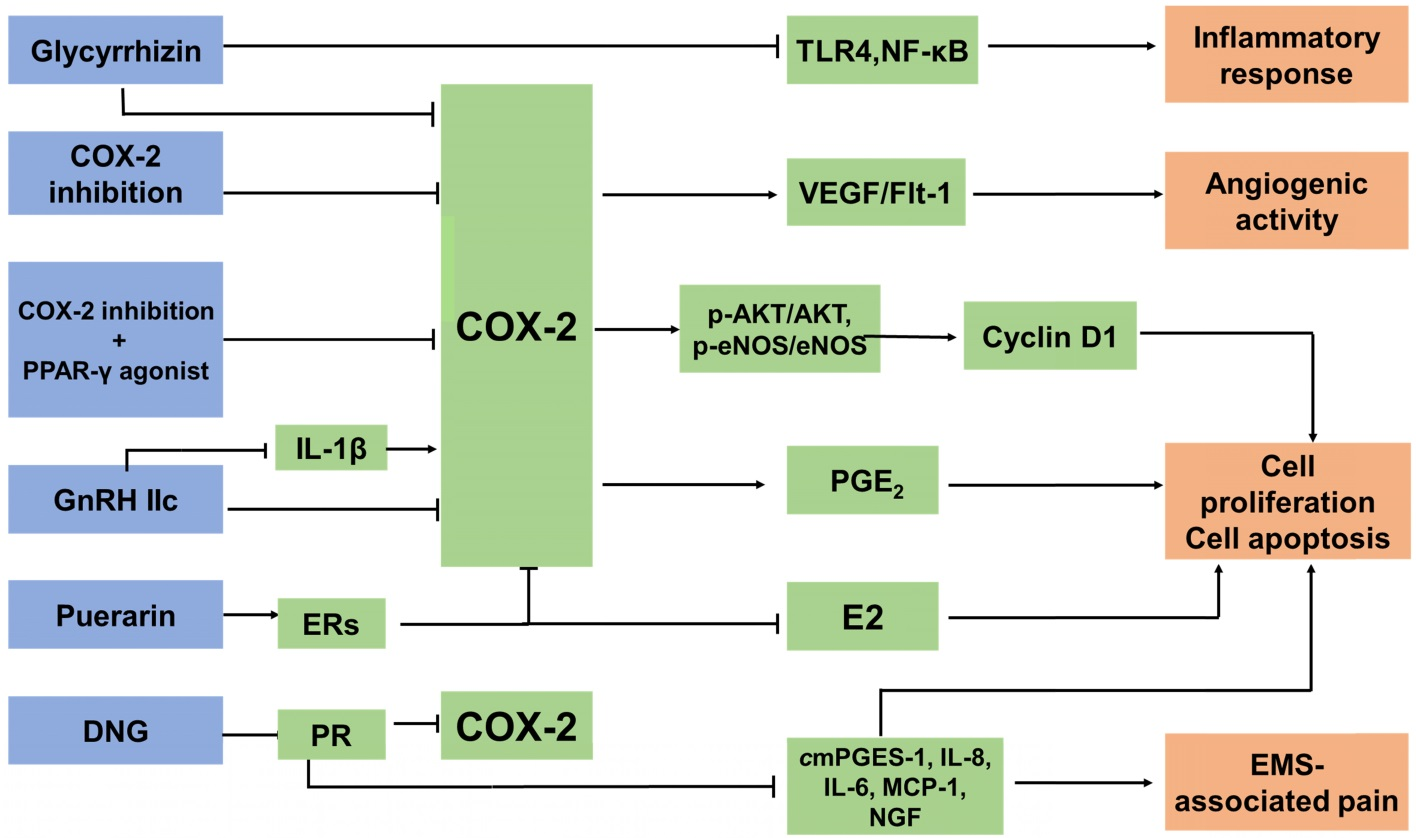
** The anti-EMS strategy of targeted COX-2.** There are three main types of anti-EMS drugs targeting COX-2: COX-2 inhibitors, hormone drugs and other drugs. They inhibit COX-2 expression in different ways. Treatment with COX‐2 inhibitors significantly decreases microvessel density and macrophage numbers, and is associated with decreased expression of VEGF and Flk-1. Combining the inhibition of COX-2 with peroxisome proliferator-activated receptor (PPAR)-γ agonists suppresses cell proliferation and induces apoptosis by decreasing the expression of p-Akt/Akt and p-eNOS/eNOS. GnRH-II decreases the COX-2 secretion of the ectopic, eutopic and normal ESC in EMS, and it can reverse the IL-1β-induced expression of COX-2 in ESCs. DNG, a selective PR agonist, downregulates the mRNA expression of CYP19A1, COX-2, mPGES-1, IL-8, IL-6, MCP-1, VEGF and NGF, and PGE2 production, as well as suppressing the development of endometriotic lesions and relieving EMS-associated pain. Glycyrrhizin is able to attenuate the expression of COX-2 and dramatically diminishes LPS-induced TLR4 expression and NF-κB activation in MEECs. As a result, it can inhibit the LPS-induced inflammatory response. Puerarin can inhibit the expression of P450arom and COX-2 in the ectopic endometrium, restrict the levels of E2 and PGE2, and block the positive feedback mechanism of E_2_ synthesis.

**Table 1:** The factors that regulates COX-2 expression in EMS

Classification	Regulatory factor	Function	Reference
**Estrogen**		hastens COX-2 expression by activated by NF-κB	Maia *et al.*2012
**Proinflammatory Cytokine**	IL-1β	stimulates the phosphorylation of ERK, p38 and JNK and results in high level of COX-2	Tamura *et al.*2002 Huang *et al.*1998
	NGF	increases *PTGS2*/COX-2 mRNA and protein levels by binding to TrkA	Wang *et al.*2009 Peng *et al.*2018
**Hypoxia**		mediates DUSP2 down-regulation, activates ERKs and MAPK, and ultimately results in the hypersensitivity of COX-2	Wu *et al.*2005 Wu *et al.*2011 Teague *et al.* 2010 Lin *et al.*2012 Pan *et al.*2007 Hsiao *et al.* 2015
**Environmental pollutants**	PCBs	plays a role in the development of endometriosis	Porpora *et al.* 2013
	HCB	activates of cytosolic AhR complex (AhR-dioxin-c-Src), triggers *PTGS2* transcription	Smith *et.al.* 1993 Deger *et al.*2007 Chiappini *et al.* 2016
**Metabolites and metabolic enzymes**	omega-3 PUFA	inhibits the activation of NF-κB and decreases the production of pro-inflammatory cytokines to reduces COX-2 expression	Tomio et al. 2013 Attaman et al.2014
	IDO	up-regulates COX-2 expression via the activation of JNK signaling pathway	Mei *et al.*2013 Mei *et al.* 2012
	LXA_4_	inhibits COX-2 expression	Kumar *et al.* 2014
**Platelets**		increases IL-1β level and increases COX-2 expression	Ding *et al.* 2015
**Others**	COUP-TFII	binds to *PTGS2* promoter to inhibit its transcription and IL-1β-induced COX-2 up-regulation	Li *et al.* 2013 Li *et al.* 2013

## Abbreviations

AA: Arachidonic acid
CNS: Central nervous system
COX-2: Cyclooxygenase-2
EMS: Endometriosis
ESCs: Endometrial stem cells
GnRH II: II-type gonadotropin releasing hormone
IL-1β: Interleukin-1β
MAPK: Mitogen-activated protein kinases
MMP: Matrix metalloproteinase
PGE_2_: Prostaglandin E_2_
VEGF: Vascular endothelial growth factor
